# Cerebral magnetic resonance elastography in supranuclear palsy and idiopathic Parkinson's disease^☆^

**DOI:** 10.1016/j.nicl.2013.09.006

**Published:** 2013-09-20

**Authors:** Axel Lipp, Radmila Trbojevic, Friedemann Paul, Andreas Fehlner, Sebastian Hirsch, Michael Scheel, Cornelia Noack, Jürgen Braun, Ingolf Sack

**Affiliations:** a Department of Neurology, Charité — University Medicine Berlin, Augustenburger Platz 1, 13353 Berlin, Germany; b NeuroCure Clinical Research Center, Charité — University Medicine Berlin, Max Delbrueck Centre for Molecular Medicine Berlin, Charitéplatz 1, 10117 Berlin, Germany; c Department of Radiology, Charité — University Medicine Berlin, Charitéplatz 1, 10117 Berlin, Germany; d Institute of Medical Informatics, Charité — University Medicine Berlin, Charitéplatz 1, 10117 Berlin, Germany

**Keywords:** MR-elastography, MRE, Elasticity, Viscosity, Parkinson disease, Progressive supranuclear palsy

## Abstract

Detection and discrimination of neurodegenerative Parkinson syndromes are challenging clinical tasks and the use of standard T_1_- and T_2_-weighted cerebral magnetic resonance (MR) imaging is limited to exclude symptomatic Parkinsonism. We used a quantitative structural MR-based technique, MR-elastography (MRE), to assess viscoelastic properties of the brain, providing insights into altered tissue architecture in neurodegenerative diseases on a macroscopic level. We measured single-slice multifrequency MRE (MMRE) and three-dimensional MRE (3DMRE) in two neurodegenerative disorders with overlapping clinical presentation but different neuropathology — progressive supranuclear palsy (PSP: N = 16) and idiopathic Parkinson's disease (PD: N = 18) as well as in controls (N = 18). In PSP, both MMRE (Δμ = − 28.8%, Δα = − 4.9%) and 3DMRE (Δ|G*|: − 10.6%, Δφ: − 34.6%) were significantly reduced compared to controls, with a pronounced reduction within the lentiform nucleus (Δμ = − 34.6%, Δα = − 8.1%; Δ|G*|: − 7.8%, Δφ: − 44.8%). MRE in PD showed a comparable pattern, but overall reduction in brain elasticity was less severe reaching significance only in the lentiform nucleus (Δμ n.s., Δα = − 7.4%; Δ|G*|: − 6.9%, Δφ: n.s.). Beyond that, patients showed a close negative correlation between MRE constants and clinical severity. Our data indicate that brain viscoelasticity in PSP and PD is differently affected by the underlying neurodegeneration; whereas in PSP all MRE constants are reduced and changes in brain softness (reduced μ and |G*|) predominate those of viscosity (α and φ) in PD.

## Highlights

• We assessed brain viscoelasticity in neurodegenerative disorders by MR-elastography.
• Parameters of brain softness and viscosity are reduced in PSP, particularly in the lentiform nucleus.
• Reduction of viscoelasticity in Parkinson’s disease is less severe and limited to brain softness.
• Reduction of brain viscoelasticity is correlated with measures of clinical severity.
• Combination of MMRE and 3DMRE is a sensitive tool to quantify regional neurodegeneration.

## 1 Introduction

Neurodegenerative disorders are defined by a progressive loss of neuronal function and structure, synaptic alteration and inflammation (reactive astrocytosis and activated microglia) (Hirsch et al., 2012). This loss of neurons and oligodendrocytes results in gross atrophy of affected brain regions, which can be reliably assessed by volumetric and morphometric measurements based on magnetic resonance imaging (MRI) (Schrag et al., 2000). In preclinical and early stages of neurodegenerative disorders, however, patterns of brain atrophy are subtle and occult to conventional MRI (Mahlknecht et al., 2010).

This is not surprising given that atrophy due to neuronal cell loss is the ultimate event in neurodegeneration. Earlier and more subtle alterations in cytoarchitecture and cellular matrix are generally missed by conventional MRI. In contrast, evaluation of mechanical properties of the brain such as elasticity and viscosity can provide information on the constitution of brain tissue at multiple scales (neuronal/non-neuronal fibre density, brain oedema and demyelination) (Posnansky et al., 2012; Riek et al., 2012; Schregel et al., 2012). Given the high sensitivity of manual palpation, the elastic response of soft tissue to controlled deformation may provide information on altered tissue architecture in disease on the macroscopic level (Sarvazyan et al., 1995).

Palpation of the brain, so far limited to neurosurgeons and pathologists to detect central nervous system disorders, has recently emerged into an imaging modality called MR elastography (MRE) (Muthupillai et al., 1995) suitable for neuroradiological examinations (Clayton et al., 2012; Green et al., 2008; Kruse et al., 2008; Pattison et al., 2010; Sack et al., 2008). In healthy volunteers, we have shown that cerebral MRE is sensitive to ageing, providing a higher sensitivity than tests using morphology-based markers (Sack et al., 2009, 2011). Furthermore, we studied the effect of multiple sclerosis (Streitberger et al., 2012; Wuerfel et al., 2010) and hydrocephalus (Freimann et al., 2012; Streitberger et al., 2011) on the viscoelastic properties of the brain and identified a disease-related loss of elasticity. Murphy et al. (2011) found a significant decrease in elasticity in seven Alzheimer patients compared to an age- and gender-matched control group without cognitive decline.

From these pilot studies we have learnt that different physiological events and various neurological disorders are accompanied by widespread softening of cerebral parenchyma, suggesting that the brain's viscoelastic properties may reflect principal patterns of neuronal integrity. To further unravel the underlying mechanisms of ‘brain softening’ in disease, recent MRE studies in the mouse investigated demyelination (Schregel et al., 2012) and inflammation (Riek et al., 2012) and identified a loss of cerebral elasticity in response to these events.

Motivated by these results, researchers are currently developing cerebral MRE into an image-based marker of neurodegeneration. However, the limited number of clinical trials still impedes conclusions on how neurodegeneration affects the brain's viscoelasticity. In particular, no comparison exists between the MRE data obtained in neurodegenerative disorders of different aetiology, clinical dynamic and cerebral distribution of the underlying neurodegeneration. Therefore, it is not possible to unambiguously correlate ‘brain softening’ with the occurrence of neuronal degradation.

For this study, we therefore chose two neurodegenerative disorders that substantially overlap in clinical presentation but differ considerably with regard to their neuropathology — progressive supranuclear palsy (PSP) and idiopathic Parkinson's disease (PD).

PD is a rather slowly progressive neurodegenerative disease with alpha-synuclein deposits in neuronal Lewy bodies and Lewy neurites as its pathological hallmark. Propagation of Lewy-related pathology (LRP) in the brainstem occurs in a caudal to rostral direction with eventual involvement of diencephalon, basal forebrain, medial temporal lobe structures and finally the cortex. Neuronal populations most vulnerable to neuronal loss in PD include the substantia nigra, locus coeruleus, raphe nuclei, pedunculopontine nucleus, basal nucleus of Meynert and dorsal motor nucleus of the vagus (Dickson et al., 2009).

In PSP, microtubule-associated protein tau is the major constituent of neurofibrillary tangles (NFTs) that accumulate in affected neurons and glial cells. Although the anatomical distribution of tau pathology determines the clinical syndrome [Williams, 2009 521/id], most PSP cases show marked atrophy of the midbrain, superior cerebellar peduncle and cerebellar dentate nucleus. Nuclei most severely affected by NFTs are the globus pallidus, subthalamic nucleus and substantia nigra. Tau pathology usually spares the cerebral cortex except for the precentral gyrus (Dickson et al., 2010).

The more rapid and widespread neurodegeneration in PSP might cause a stronger reduction in brain elasticity compared with the rather limited neuronal loss within the substantia nigra in patients with early to moderate PD.

To prove this hypothesis we measured and analysed externally induced shear vibrations in the brain using 2D- and 3D-MRE. We used fast 2DMRE to capture the shear modulus at multiple drive frequencies, coining the term multifrequency MRE or MMRE, and 3D-MRE to measure the full vector field of the vibration at single frequency (3DMRE). Each method represents a trade-off between acquisition time and completeness of elastodynamic information. 3DMRE offers an opportunity to perform a detailed mapping of viscoelastic parameters while MMRE enables us, for regions of interest (ROI's), to extend the analysis in terms of modelling. Under the assumption of scale-free viscoelastic network topology in the brain, MMRE offers greater interpretative power (Sack et al., 2013) while under different assumptions (e.g. dominating elastic properties) MMRE provides equivalent measures to 3DMRE at single vibration frequency. We therefore aimed to use both methods for a detailed analysis of the effect of neurodegenerative diseases in MRE.

Several clinical studies of brain MRE are based on MMRE in combination with the so-called springpot model (Freimann et al., 2012; Streitberger et al., 2011, 2012; Wuerfel et al., 2010). The springpot provides two constants, μ and α. μ corresponds to our haptic sensation of a material's constitution (related to the terms ‘soft’ and ‘firm’), while α is the viscoelastic power law exponent and relates to the density and topology of the mechanical lattice (Posnansky et al., 2012). Other studies report 3DMRE of the brain measured at single frequency (Green et al., 2008; Murphy et al., 2011). Consistent with recent studies in the mouse, we will state for this type of data the magnitude (|G*|) and the phase angle (φ) of the complex shear modulus (Schregel et al., 2012). Due to its capability to efficiently suppress pressure waves, 3DMRE can provide higher spatial resolution than MMRE, however, viscoelastic modelling (and drawing conclusions about the underlying mechanical network) requires multiple vibration frequencies as used in MMRE. We therefore apply both experiments with a view to using MRE for the staging of neurodegeneration.

## 2 Methods

### 2.1 Patients

The study was approved by the Institutional Review Board and all subjects gave written informed consent before participation.

The study cohort included 52 subjects, among them 18 patients (6 female; mean age = 63 years) diagnosed with mild to moderate Parkinson's disease according to the UK Brain Bank consensus criteria (Hughes et al., 1992), 16 patients (8 female; mean age = 70 years) diagnosed with probable PSP according to current consensus criteria (Litvan et al., 1996, 2003) and 18 predominantly sedentary control subjects of similar age (8 female; mean age = 64 years) (Table 1). As clinical heterogeneity of PSP weakens diagnostic certainty, recruitment of PSP patients was limited to the clinical phenotype of Richardson's syndrome (PSP-RS) and PSP-Parkinsonism (PSP-P) (Williams and Lees, 2009). Subjects were recruited from the Outpatient Movement Disorder Unit of the Charité, Berlin, and the NeuroCure Clinical Research Center. Subjects with implanted deep brain stimulators (STN-DBS) or carrying other ferromagnetic implants were not included. The presence of structural brain abnormalities in T1- and T2-weighted MRI unrelated to PSP/PD such as birth defects, head trauma and cerebrovascular disorders excluded subjects from further participation. Clinical severity of underlying neurodegeneration was rated using appropriate test instruments. In PD, the motor part of the Unified Parkinson's Disease Rating Scale (UPDRS part III) was obtained during a phase of best medical treatment (ON state). In PSP, disease severity was assessed by the Golbe scale (PSPRS) (Golbe and Ohman-Strickland, 2007).

### 2.2 MRE measurements

Mechanical vibrations were transmitted into the head by a custom-made head cradle connected via a carbon-fibre piston to a remote vibration generator as described in (Sack et al., 2008). Measurements were performed on a standard 1.5 T clinical MRI scanner (Sonata, Siemens, Erlangen, Germany) equipped with a single-element head-coil. For both MMRE and 3DMRE, a single-shot spin echo echo-planar imaging sequence was employed, which was sensitized to motion by a sinusoidal motion-encoding gradient (MEG) during the first half of the echo period.

#### 2.2.1 Single-slice multifrequency MRE (MMRE)

The vibration waveform was synthesised by superposition of four harmonic oscillations of f = 25, 37.5, 50 and 62.5 Hz frequency with identical phases and a total duration of 400 ms (Klatt et al., 2007). A single burst of this signal was fed into the wave generator prior to the start of each image acquisition. The motion was encoded by an MEG in through-plane direction composed of four sinusoids of 60-Hz frequency and 35 mT/m amplitude. The polarity of the MEG was toggled in each second experiment for subtracting the inverse phase contrast and leaving the difference wave phase in the image. The experiment was repeated in order to capture the dynamics of wave propagation. Therefore, the delay between the onset of vibration and the start of motion encoding was varied 32 times from 320.0 ms to 397.5 ms by an increment of 2.5 ms. The resulting phase shift corresponds to a first harmonic frequency of 12.5 Hz, which determines the resolution in our vibration spectrum. One 6-mm transverse image slice through the central part of the ventricles parallel to the internal base of the skull was selected. Further image acquisition parameters were: repetition time TR, 3 s; echo time TE, 149 ms; field of view, FoV, 192 × 192 mm^2^; matrix size, 128 × 128; no accumulation.

#### 2.2.2 Three-dimensional MRE (3DMRE)

3DMRE refers to full wave field acquisition within a volumetric slab of 6 cm thickness through the central brain. Continuous harmonic vibrations of 50 Hz frequency were used for head stimulation in this experiment. The strain wave field was consecutively encoded by an MEG in through-plane direction, phase-encoding direction and read-out direction, each composed of three cosine-cycles of 60-Hz frequency (zeroth- and first-moment-nulled MEG (Murphy et al., 2011)). The cosine-shaped gradient waveform was approximated by trapezoidal gradients of 30 mT/m amplitude. Four instances of one vibration cycle were captured by a trigger-shift increment of 5 ms. Thirty transverse slices of 2-mm thickness without gap were acquired in the central cranium parallel to the genu splenium axis of the corpus callosum. Further imaging parameters were: TR, 272 ms; TE, 116 ms; FoV, 256 × 224 mm^2^; matrix size, 128 × 122; two accumulations for increasing the signal-to-noise ratio.

### 2.3 Data processing

Phase images were unwrapped and Fourier-transformed in time, yielding complex displacement fields at drive frequency. The wave field maps were filtered either by applying the curl operator followed by a 3D Gaussian noise filter to a 5-pixel neighbourhood (for 3D data) or by a 2D Butterworth band-pass with frequency-dependent filter threshold given in Klatt et al. (2007) (for multifrequency 2D data). While the preprocessing of 2D data corresponds to our previously published method, 3D processing benefits from the capability of the curl operator to suppress compression waves which is not applicable to 2D data. Modulus recovery was based on a pixel-wise inversion of the Helmholtz equation as analysed in Papazoglou et al. (2008) assuming a uniform density of brain tissue of 1000 kg/m^3^.

#### 2.3.1 MMRE

For 2D data of MMRE, the real part and the imaginary part of G* were averaged over the parenchyma visible in the image slice (excluding the ventricles), yielding four global frequency-dependent complex modulus values G*(f) with f being the drive frequency. These shear moduli were fitted by the springpot model,

(1) $$G*=\kappa {\left(i\cdot 2\pi \cdot f\right)}^{\alpha },$$

where κ = μ^1 − α^η^α^, and κ and α were the frequency-independent free variables in our least-squares fit procedure. The parameter μ is the global shear elasticity; η is the viscous damping and α is a measure of the elastic lossy relation (Sack et al., 2009). For example, α = 0 corresponds to lossless elastic behaviour with shear elasticity, μ and α = 1 to lossy viscous damping with viscosity, η. For relating κ to a shear elasticity μ, we assumed η = 3.7 Pa·s. This value of η was previously determined as an approximated value of viscosity in human brain tissue (Klatt et al., 2007).

#### 2.3.2 3DMRE

For 3D data, each field component was separately inverted, yielding three complex shear modulus maps, which were combined to generate one complex shear modulus map G* represented as

(2) $$\begin{array}{l} \left|G*\right|=\mathrm{abs}\left(G*\right)\\ \varphi =\arctan\left(\mathrm{imag}\left(G*\right)/\mathrm{real}\left(G*\right)\right). \end{array}$$

This representation of G* was chosen for comparing the phase angle φ to the springpot-related constant α which will be discussed later. Additionally, negative G″-values due to reversely running waves could be eliminated in this way. For completeness, the global values of real(G*), imag(G*), |G*| and φ in the parenchyma excluding the ventricles are tabulated. For comparison of |G*|- and φ-parameter maps, one central image slice through the genu and splenium of the corpus callosum was manually selected for each subject and registered to a template generated from the MRE magnitude images of all subjects using the ANTs open source software library (Avants et al., 2011). The transformation model used in our registration was normalised symmetric (Greey SyN) probability mapping.

### 2.4 Statistical analysis

All data are expressed as means ± SD. Parameters of brain viscoelasticity (|G*|, φ) were calculated for both, the full brain and the area of the basal ganglia (lentiform nucleus: putamen, internal and external globus pallidus), and compared by ANOVA. Groups (subjects vs. patients) were compared using unpaired t-test (parametric data) or Mann–Whitney test (nonparametric data). As age is an important determinant of brain elasticity, ANOVA was performed with age as a covariate. Correlation analysis between clinical data (age, disease duration, disease severity) and parameters of brain elasticity was calculated by Pearson's correlation coefficient. *P* < .05 was considered statistically significant. All calculations were performed using GraphPad Prism Version 5.01 (GraphPad, Inc., La Jolla, CA, USA). Owing to the exploratory nature of this pilot study, no comparisons for multiple testing were made.

## 3 Results

### 3.1 Clinical characteristics

Clinical characteristics of cases and controls are summarised in Table 1. Among PD cases, eight had an akinetic-rigid phenotype, three were tremor dominant and seven had an equal symptom presentation. Among PSP cases, eight met criteria for Richardson subtype and eight for Parkinson subtype of PSP (PSP-P). Disease duration was slightly shorter (*P* = 0.28) in PSP, reflecting the faster progression of PSP, and there was a trend (*P* = 0.07, ANOVA) towards an older age in PSP patients (+ 6 years compared to controls).

### 3.2 Brain viscoelasticity in neurodegeneration

Age has been reported to be a determinant of brain viscoelasticity, accounting for a linear decline in whole brain elasticity (μ) of − 0.75%/year (Sack et al., 2011), whereas tissue's microstructure (α) remains unchanged. To separate these age-related changes from the impact of neurodegeneration on brain viscoelasticity, statistical comparisons of MRE parameters included age as covariate. For group-wise comparisons, 3DMRE parameters obtained in PSP cases were corrected by − 0.75%/year.

**Fig. 1:**
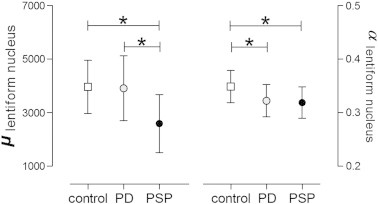
Group-wise comparison of MMRE parameters μ and α within the lentiform nuclei; values are group means [SD], *P < 0.05, unpaired t-test.

When compared to control subjects, no significant change in whole brain MMRE parameters μ and α was found for PD. In contrast, PSP was associated with a significant reduction of both μ and α of − 28.8% (vs. controls: *P* < 0.001) and − 4.9% (vs. controls: *P* < 0.01), respectively. This effect was pronounced in the lentiform nucleus (vs. controls: Δμ = − 34.6%, *P* = 0.001; Δα = − 8.1%, *P* < 0.01). Considering this region in PD patients, only a weak reduction of α of − 7.4% (vs. controls: *P* < 0.05) was discernible, while μ remained unchanged (Fig. 1).

3DMRE reproduced our primary findings of stronger reduction of viscoelastic constants in PSP compared to PD (Δ|G*|: − 10.6%, *P* < 0.01 [PSP vs. controls]; − 4.8%, not significant [PD vs. controls]; Δφ: − 34.6%, *P* < 0.001 [PSP vs. controls]; − 15.4%, *P* = 0.07 [PD vs. controls]) and pronounced reduction of MRE parameters in the lentiform nucleus (Δ|G*|: − 7.8%, *P* = 0.037 [PSP vs. controls]; − 6.9%, *P* < 0.05 [PD vs. controls]; Δφ: − 44.8%, *P* < 0.001 [PSP vs. controls]; − 20.7%, *P* = 0.06 [PD vs. controls]) (Fig. 3).

**Fig. 2:**
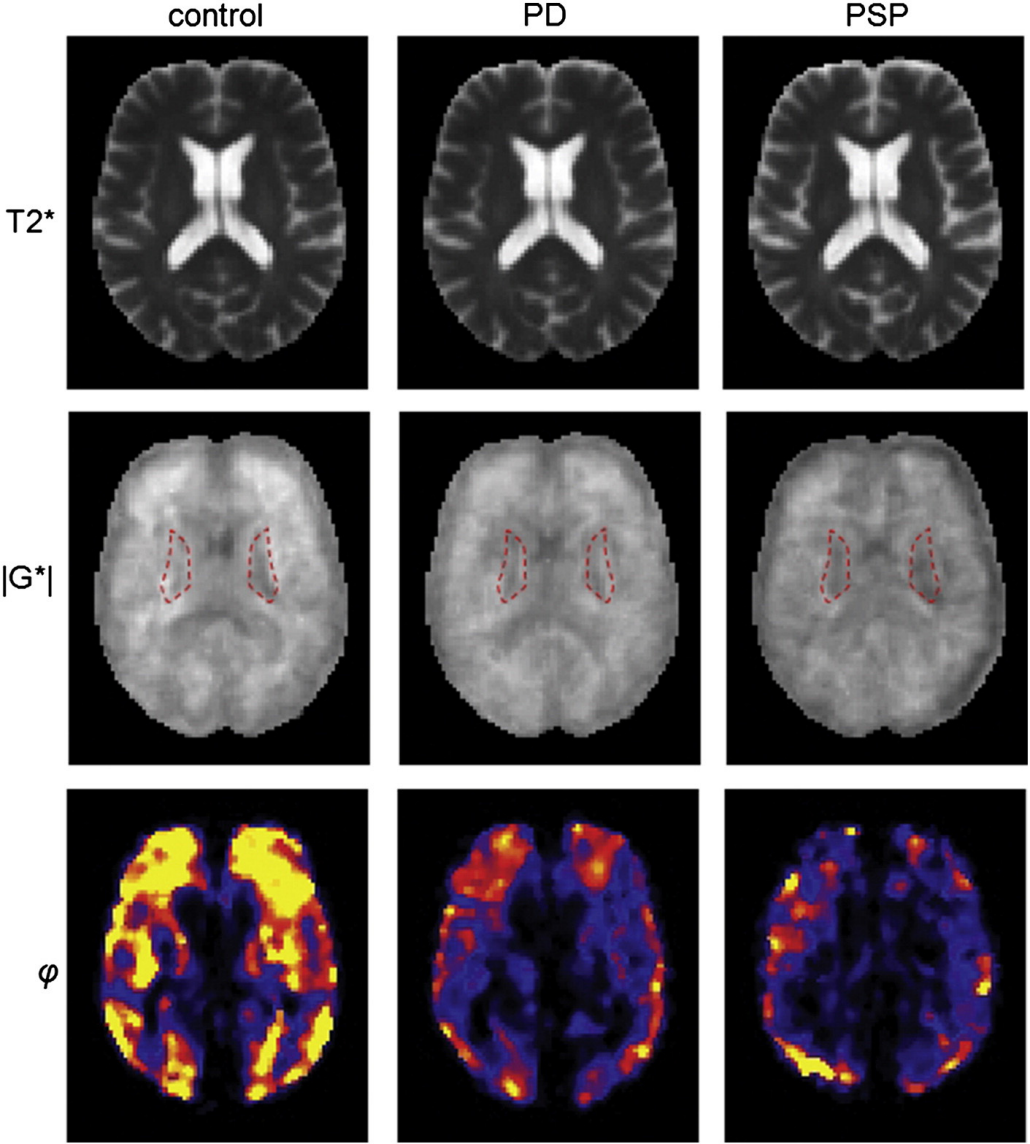
Normalised parameter maps obtained by 3DMRE. The grayscale in the |G*| maps is from 0 to 3 kPa, the colorscale of phi is from 0 to 0.2. (For interpretation of the references to colour in this figure, the reader is referred to the web version of this article.)

**Fig. 3:**
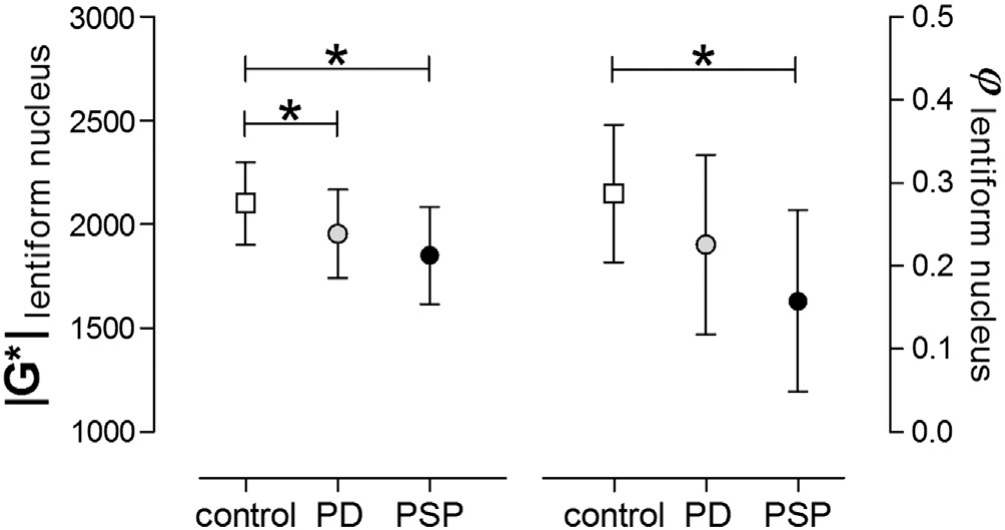
Group-wise comparison of 3DMRE parameters |G*| and φ within the lentiform nuclei; values are group means [SD], *P < 0.05, unpaired t-test.

Contrary to MMRE, where Δμ > Δα, in 3DMRE Δ|G*| < Δφ, i.e. the dimensionless phase-based parameter, displayed a higher disease-related change than the shear-modulus parameter, highlighting that the mechanical constants measured by MMRE and 3DMRE provide independent information on brain constitution. Although μ and φ display similarly high rates of change with disease, φ has a much higher intra-group variability and is thus less reliable than μ. The high variability of φ is also reflected in the normalised parameter maps shown in Fig. 2 for |G*| and φ in a central slice of each of our groups. Fig. 2 addresses the local variation of 3DMRE parameters. Since φ reflects the duality of fluid–solid properties of tissue it is highly affected by the heterogeneous distribution of fluid-filled spaces in the brain. In contrast, |G*| appears to be smoother in the normalised group maps with less in-plane variation than φ, which is consistent with the relative magnitude of the standard deviations given in Table 2. Both |G*|- and φ-image intensities decrease from the healthy state to PD and PSP. Again, pronounced signal deterioration is seen in the lentiform nucleus, which are demarcated by dashed red lines in the |G*| maps in Fig. 2. Mean intensities and SD values in these regions are 1913 ± 196 Pa, 1757 ± 117 Pa, 1551 ± 140 Pa for controls, PD and PSP patients, respectively. From 2D-MMRE no normalised parameter maps were attainable. All group mean values and standard deviations are summarised in Table 2.

### 3.3 Correlation of brain viscoelastic properties with clinical data

**Fig. 4:**
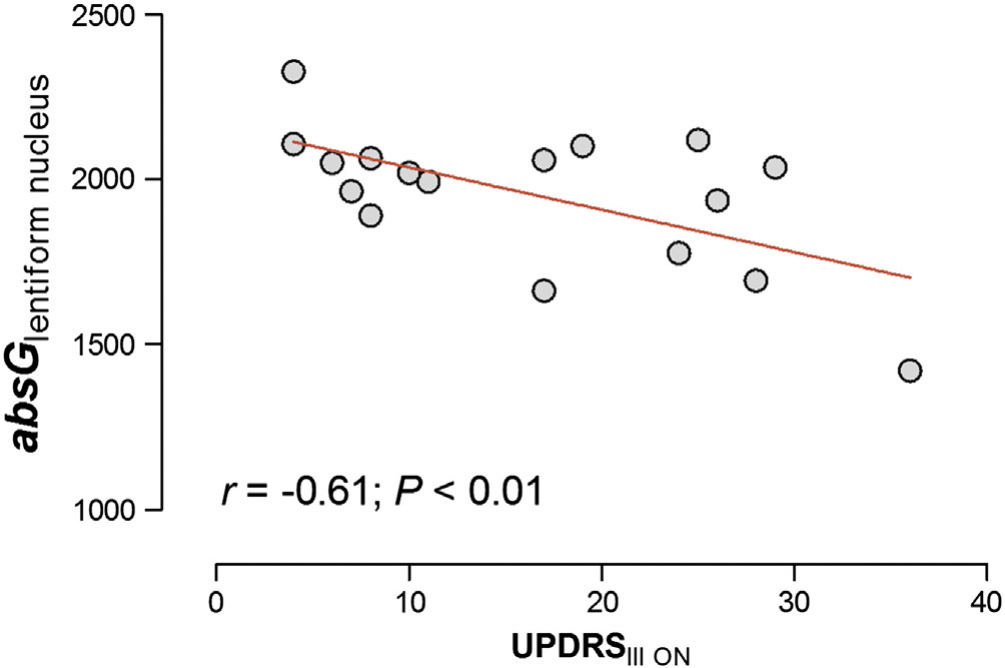
Correlation of 3DMRE (|G*|) and severity of clinical symptoms (UPDRS_III-ON_) in PD patients.

The impact of neurodegeneration on brain viscoelastic properties also becomes apparent when disease severity and elasticity parameters are correlated (Table 3, Fig. 4). In the present study, patients had mild to moderate PD with a mean UPDRS_III-ON_ of 16.7 pts., ranging from 4 to 36 pts. There was a strong correlation between UPDRS_III-ON_ and 3DMRE parameters obtained both in the full brain and in the lentiform nucleus (all *r* < − 0.5, all *P* < 0.05, Fig. 4). In PSP, 3DMRE parameters correlated with disease stage (PSP staging system, full brain and lentiform nucleus, all *r* < − 0.5, all *P* < 0.05 [except *imagG*]) and less robustly with the clinical symptom score (Golbe score vs. φ_full brain_: *r* = − 0.51, *P* = 0.04).

Direct comparison of MMRE parameters between cases shows a significant reduction of μ in PSP patients (PD vs. PSP: full brain Δμ = − 35.6%, *P* < 0.001; lentiform nucleus Δμ = − 36.7%, *P* < 0.001), reflecting the more rapid and widespread neurodegeneration. Group-wise comparison of 3DMRE parameters (PD vs. PSP), however, did not reach statistical significance (PD vs. PSP: full brain Δφ = − 22.0%, *P* = 0.058; lentiform nucleus Δφ = − 30.0%, *P* = 0.08).

As previously discussed, age is a known determinant of brain viscoelasticity (Sack et al., 2009, 2011). Accordingly, there was a strong negative correlation between age and all 3DMRE parameters in PD (full brain and lentiform nucleus, *r* = − 0.49 to − 0.76, all *P* < 0.05 [except φ_full brain_]). In contrast, age dependency was less distinct in controls (*imagG*_full brain_ *r* = − 0.47, *P* = 0.048) and non-significant in PSP cases, probably due to the smaller age range in these groups (PD: 32 to 77 [∆45] years, controls: 49 to 86 [∆37] years, PSP: 58 to 83 [∆25] years). Contrary to 3DMRE, correlation of MMRE parameters with any of the clinical data (age, severity, or stage) was poor. Neither of the two groups showed a correlation between disease duration or gender and MRE parameters.

## 4 Discussion and conclusion

### 4.1 Group wise comparison

Our study addressed the alteration of brain viscoelastic constants in two clinically similar but neuropathologically distinct neurodegenerative conditions.

The main results of our study are as follows: (1) brain viscoelasticity is reduced in PSP, with a greater reduction within the lentiform nuclei; (2) reduction of brain viscoelasticity is highly correlated with measures of clinical severity in both, PSP and PD; and (3) reduction of viscoelasticity in PD is limited to measures of softness (μ, |G*|), while in PSP measures of viscosity (α, φ) are affected as well.

To date, standard T_1_- and T_2_-weighted cerebral MRI (1.5 T) is insufficient in detecting PD, especially at early stages (Seppi and Schocke, 2005), and thus is primarily used to exclude potential cases of symptomatic PD. Midbrain and tegmental atrophy as well as frontal and temporal lobe atrophy have been proven to reliably discriminate PSP from PD and control subjects; however, specificity against atypical Parkinson syndromes (multiple system atrophy, corticobasal syndrome) is poor (Lee et al., 2005).

Advanced quantitative structural MR-based techniques such as MRE and diffusion tensor imaging (DTI) provide more specific measures of the cellular matrix of the brain parenchyma and thus improve the classification sensitivity/specificity for neurodegenerative disorders (Menke et al., 2009). As shown in the present study and in our previous work (Sack et al., 2011), normal ageing is accompanied by a linear decline in whole brain elasticity as shown by a decrease in μ and |G*|. This is supported by DTI, where fractional anisotropy (FA), a measure of white matter connectivity, decreases linearly after the second decade of life (Lebel et al., 2012; Sullivan et al., 2010).

In neurodegenerative disorders such as PSP, both measures (FA Whitwell et al., 2011 and MRE) are significantly decreased compared to healthy age-matched controls, indicating progressive degradation of the brain cellular matrix. Unlike reduced FA in DTI, the MRE results of the present study indicate that neurodegeneration in PSP involves at least two distinct processes — progressive loss of brain elasticity (reduced μ and |G*|) and reduction of the viscous damping properties of the brain (reduced α and φ). The physical quantity underlying DTI is the water diffusion coefficient. This coefficient is correlated with the displacement of diffusing water, which is indirectly related to the directionality and integrity of the underlying tissue structure. Due to the scaling properties of viscoelastic constants in hierarchically ordered tissue (Kelly and McGough, 2009), MRE provides a more direct measure of the inherent constitution and the microstructure of the tissue under investigation (Guo et al., 2012; Posnansky et al., 2012).

### 4.2 Comparison of 2D and 3D-MRE

Before discussing our results with respect to the underlying pathophysiology we wish to comment on the viscoelastic notation used in this study. The classic measure in MRE is the complex shear modulus, which has a real and an imaginary part, also known as storage modulus (G′) and loss modulus (G″), respectively. Both parameters are translated to frequency-independent, i.e. generalised, constants by the springpot model (Eq. (1)), which is well-established in the MMRE literature (Asbach et al., 2010; Klatt et al., 2010; Sack et al., 2009). The springpot implies a constant ratio of G″/G′ and constant slopes of G″ and G′ in logarithmical coordinates. The ratio is related to our parameter α by α = 2/π arctan(G″/G′). Furthermore, α is identified as the slope of logG′(logω) and logG″(logω) (Klatt et al., 2010). Thus, our 3DMRE parameter φ = arctan(G″/G′) (see Eq. (2)) should equal π/2 · α, provided that the simple two-parameter springpot model is valid within our frequency range (from 25 to 62.5 Hz). As this is not fully true (see e.g. Fig. 3 in Sack et al., 2009), we cannot compare φ with α. A further obstacle to comparing the two phase-based parameters φ and α is their numerically different treatment. The calculation of α invoked spatially averaged G′- and G″-values followed by model-fitting. In contrast, φ was derived from G′- and G″-maps and registered to normalised images as shown in Fig. 2. Consequently, α is less prone to noise than φ, rendering α more reliable for the assessment of global viscoelastic effects. The relationship between μ and |G*| depends on α and is thus more complex. μ and |G*| can be considered equivalent only in materials with dominating elastic properties. Although brain tissue is more elastic than viscous (Klatt et al., 2007), |G*| is influenced by viscosity, which may explain its lower rate of change upon disease. At any rate, a decline of μ (and of |G*| in elastic solids) indicates ‘softening’, whereas the decay of α or φ suggests transition to a more elastic material (Guo et al., 2012; Posnansky et al., 2012).

Softening with unchanged α would imply that the architecture of the tissue remains preserved while its mechanical scaffold becomes weaker. Recent studies on isolated cells (Lu et al., 2006) and in vivo murine brain (Schregel et al., 2012) indicate that axons represent important constitutive elements of the mechanical scaffold of the brain. Schregel et al. (2012) observed a drop in |G*| in the presence of extra-axonal reorganisation, i.e. demyelination and degradation of the extracellular matrix similar to observations made by Riek et al. (2012) in a mouse model of neuroinflammation. Since these processes do not affect the topology of axonal fibres, its influence on α is presumably low, which is consistent with our previous findings in mild (remitting-relapsing) MS (Wuerfel et al., 2010) and in the maturating brain (Sack et al., 2009). Interestingly, for progressive MS (primary and secondary progressive, pp&sp) and normal pressure hydrocephalus (NPH), an MMRE parameter decrement on the same order as in our PSP group was reported (MS [pp&sp]: Δμ = − 20.5%, Δα = − 6.1% (Streitberger et al., 2012); NPH: Δμ = − 25.1%, Δα = − 9.5% (Streitberger et al., 2011)).

In the light of these results, a drop in |G*| and μ without an unchanged parameter α suggests the presence of processes like inflammation or disruption of extra-axonal integrity whereas progressive degradation towards neuronal loss would ultimately cause a decline in α as has been observed in progressive MS, NPH (Streitberger et al., 2011, 2012) and in the PSP group of our current study.

With our current knowledge, we can only tentatively interpret the different patterns of brain viscoelastic changes in PD and PSP. The neuropathology of PD involves presynaptic accumulation of α-synuclein (Cheng et al., 2010; Schulz-Schaeffer, 2010), which starts focally and affects axonal integrity only later in the process of degeneration. In PSP, hyperphosphorylated tau dissociates from microtubules, causing disruption of microtubular transport and eventually axonal degradation (Armstrong and Cairns, 2013). Thus, early loss of axons that are eminent to the mechanical scaffold of the brain (Freimann et al., in press) might explain the pronounced loss of |G*|, μ and α in our group of PSP patients, while unchanged MRE parameters indicate that axonal degradation is probably not the primary pathological mechanism in PD. Varying degrees of extraneuronal involvement (glial, astrocytes) in PSP and PD might contribute to the pronounced reduction of MRE parameters in our PSP cases. Although there is neuropathological evidence of limited glial α-synuclein aggregates also in PD (Fellner et al., 2011), tau pathology is dominant in oligodendroglia and astrocytes in PSP (Armstrong and Cairns, 2013), altering the mechanical scaffold of the brain even further.

### 4.3 Limitations

The link of MRE parameters to histological properties of brain tissue is still controversial and needs further verification. Precision of the phase angle of the complex modulus (φ) is limited, which prevents us from drawing further conclusions about the sensitivity of cerebral MRE to neuronal network structures.

Some technical matters concerning the combination of MMRE and 3DMRE remain to be addressed. In our study, MMRE and 3DMRE had to be applied separately due to time constraints. A combined method of 3DMMRE appears feasible with the aid of 3 T MRI and parallel imaging. 3DMMRE would combine the sensitivity of μ with the capability of 3DMRE to provide spatially resolved parameter maps. New developments in MRE reconstruction methods would largely benefit from 3D wave data at multiple drive frequencies (Baghani et al., 2011; Papazoglou et al., 2012; Van Houten et al., 2011).

Our study has several limitations. First of all, brain viscoelasticity is known to be inversely related to age. Therefore, the non-significant trend towards a younger age among our PD patients might overestimate the differences in MRE between both groups. The effect of age on MRE parameters, however, was non-significant in our PSP patients and only limited (*imagG*_full brain_) in our control group. Second, MRE results in our PD patients varied widely. This is explained in part by a large age range (32–77 years) and wide differences in clinical severity (UPDRS_III-ON_: 4–36 pts.), parameters that showed the highest impact on the brain's viscoelastic properties. Future studies assessing MRE longitudinally in neurodegenerative disorders such as Parkinson's disease, multiple system atrophy and PSP will help to define diagnostic thresholds for an image-based differentiation of neurodegenerative diseases.

In summary, 3DMRE for spatially resolved mechanical parameter mapping and MMRE for viscoelastic modelling were applied to the brains of patients with PD and PS and compared to controls. Both MRE methods revealed a reduction of whole-brain elasticity and viscosity in PSP due to widespread neurodegenerative processes but showed no alteration of global viscoelasticity in PD. However, regional analysis by 3DMMRE showed that PD affects the basal ganglia region causing softening of the tissue. Overall, MMRE was sensitive enough to discriminate PSP from PD based on the global shear modulus while the enhanced regional sensitivity of 3DMRE provided the highest correlation with clinical scores in PD. In the future, a combination of MMRE and 3DMRE may provide a highly sensitive imaging marker for the quantification of regional neurodegeneration and the distinction of different types of neurodegenerative disorders.

## Figures and Tables

**Table 1:** Patient characteristics.

Parameter	Controls	PD	PSP	*P*
N	18	18	16	
Gender [f: m]	8: 11	6: 12	8: 8	
Age [years]	64 ± 10.8	63 ± 10.8	70 ± 5.8	0.07^a^
Disease duration [months]	n.a.	111 ± 81.0	69 ± 32.4	0.14^b^
Clinical severity^c^ [points]	n.a.	16.7 ± 9.8	41.9 ± 12.5	

Values are means ± SD.

a Kruskal–Wallis test.

b Mann–Whitney test.

c Clinical severity in PD and PSP cases assessed by motor part of UPDRS and Golbe scale.

**Table 2:** Brain viscoelastic parameters.

Viscoelasticity parameters	Controls	PD	PSP	*P*^+^
MMRE — full brain				
μ [Pa]	2788 ± 302	3038 ± 814	1984 ± 489⁎,⁎⁎	< 0.001
α	0.303 ± 0.014	0.295 ± 0.018	0.288 ± 0.012⁎	0.02
MMRE — lentiform nucleus				
μ [Pa]	3961 ± 997	3907 ± 1211	2475 ± 1036⁎,⁎⁎	< 0.001
α	0.349 ± 0.03	0.322 ± 0.03⁎	0.319 ± 0.029⁎	0.01
3DMRE — full brain				
realG* [Pa]	1814 ± 155	1737 ± 211	1574 ± 145⁎	< 0.01
imagG* [Pa]	588 ± 94	525 ± 143	423 ± 92⁎	< 0.01
|G*| [Pa]	1970 ± 176	1876 ± 255	1682 ± 170⁎	< 0.01
φ	0.26 ± 0.04	0.22 ± 0.07	0.17 ± 0.07⁎	0.01
3DMRE — lentiform nucleus				
realG* [Pa]	1942 ± 182	1804 ± 180 ^+^	1745 ± 213 ^+^	0.05
imagG* [Pa]	620 ± 129	530 ± 158	437 ± 128 ^+^	< 0.01
|G*| [Pa]	2101 ± 199	1955 ± 213 ^+^	1850 ± 233 ^+^	0.01
φ	0.29 ± 0.08	0.23 ± 0.11	0.16 ± 0.11 ^+^	< 0.01

Values are means ± SD; ^+^1-way ANOVA (age as covariate); post hoc between-group analysis (unpaired t-test).

⁎ *P* < 0.05 vs. controls.

⁎⁎ *P* < 0.05 vs. PD.

**Table 3:** Correlation of MRE parameters and clinical severity (PD: UPDRS motor part during ON; PSP: PSP staging system according to Golbe scale (Golbe and Ohman-Strickland, 2007)).

	PD *r*	PD *P*	PSP *r*	PSP *P*
MMRE — full brain				
μ	0.030	0.907	− 0.285	0.285
α	− 0.376	0.124	− 0.431	0.095
MMRE — lentiform nucleus				
μ	0.487	0.041	− 0.259	0.334
α	− 0.068	0.790	− 0.153	0.570
3DMRE — full brain				
realG*	− 0.592	0.010	− 0.536	0.032
imagG*	− 0.582	0.011	− 0.503	0.047
|G*|	− 0.589	0.010	− 0.540	0.031
φ	− 0.533	0.023	− 0.500	0.048
3DMRE — lentiform nucleus				
realG*	− 0.593	0.012	− 0.511	0.043
imagG*	− 0.486	0.048	− 0.420	0.105
|G*|	− 0.607	0.010	− 0.506	0.046
φ	− 0.478	0.053	− 0.548	0.028
